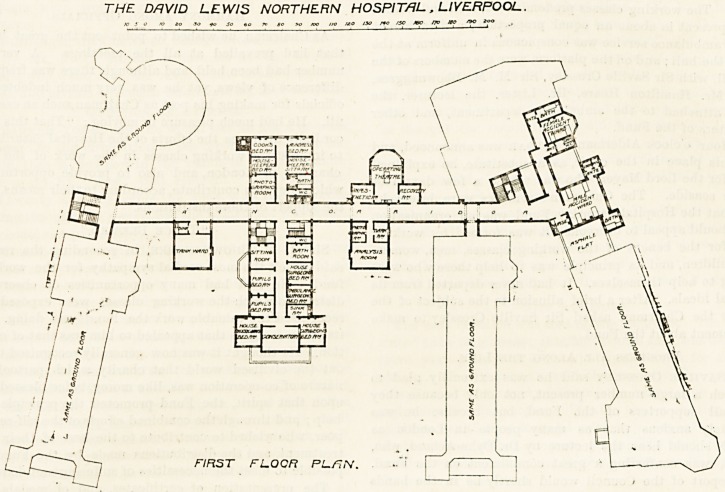# The David Lewis Hospital, Liverpool

**Published:** 1902-03-29

**Authors:** 


### THE INSTITUTIONAL WORKSHOP.

### THE DAVID LEWIS HOSPITAL, LIVERPOOL.

THIS hospital, recently opened, owes its existence to the generosity of the late David Lewis, and to his trustees who placed a sum of £120,000 at the disposal of the governors. The original hospital contained only 23 beds. It was situated in Back Leeds Street, and was soon found to be quite inadequate. A much larger building was erected in 1845, in Great Howard Street, but even this was insufficient for the growing wants of the neighbourhood, and the hospital was repeatedly added to. Latterly, to find room for more female patients, it was necessary to place the nursing staff in another building. Evidently some further step was imperative to increase the size of the institution, and the old hospital formed no exception to the general rule in buildings more than a generation old—it was declared insanitary. The munificence of the David Lewis Trust freed the governors from all anxiety and the present fine hospital is the outcome of that munificence and of the time and watchfulness of the governors. The site consists of about 12,000 square yards, and has its most extensive frontage to Great Howard Street, being bounded on the north by the Lancashire and Yorkshire Railway, and on the south by Leeds Street. It is on the west, or Great Howard Street front, that the main entrance to the administrative block is placed. Reference to the plan will show that this block projects westward from a corridor running north and south. It contains vestibules, waiting-room, committee-room, and resident medical officer's rooms. East of the corridor are the store-rooms, and these are close to the laundry. A little further along the corridor to the south are the lecture-room and museum, and on the north side of the administrative block is a small room named the tank ward, for the treatment of enteric fever patients, and on the opposite side are the matron's rooms.

The north boundary of the site is occupied by two blocks, the most easterly of which is a circular block containing the children's wards for 17 beds on each floor. These wards are suitably decorated, and the roof is flat and is intended as a playground. The sanitary block is conveniently placed and correctly designed. The main staircase is placed between the children's block and the block to the west of the children's wards. The west block contains, on the ground floor, a ward for 22 beds. It is 96 feet long, 26 feet wide, and 13 feet high, giving about 1,500 cubic feet per patient. The ward adjuncts are a sun-room, verandah, a pay ward, apparently for one bed, an isolation ward, a bath-room, kitchen, small day-room, and a sanitary block correctly placed and designed. Generally it may be said that the whole block is well designed, and the large ward itself exceptionally good; but we should like to have seen a nurses' duty-room, where the bath-room is, and the bath-room occupying the position of the sun-room, the latter being transferred to the verandah. The isolation ward should have been thrown out about six feet beyond the other rooms, so obtaining space for two windows and direct cross-ventilation. The same remark applies to the pay ward. It may be that both these rooms have fanlights over their doors. This arrangement is worth something, but it is only ventilation into a corridor, and not to the open air. The first-floor plan is the same as the ground plan, and the south block is the same as the north.

The warming is apparently by open fireplaces in the centre of the wards. This system entails a horizontal flue,

THE DAVID LEWIS NORTHERN HOSPITAL, LIVERPOOL.

GROUND FLOOR PLAN.

and the horizontal flue generally entails a granolith or terrazzo floor, which in its turn entails a thick layer of concrete, and in spite of all cracking of the terrazzo often occurs.

The large block, which stretches along the south boundary, is composed of the nurses' home and of the out-patients' department. These are continuous. The out-patients' department has a fine waiting hall, and around it are placed the consulting-rooms, dressing-rooms, operating room, dispensary, and the closets. It is curious to notice that the latter are not cut off from the block by a ventilating passage, and this peculiarity applies also to the closets in the administrative block.

The nurses' home can be approached from the main corridor or from the Leeds Street entrance. On the right of the latter is the receiving casualty room and the hall porter's room. Turning to the west, and passing through a conservatory, the nurses' rooms are reached. They are divided by a corridor running east and west and ending in an octagonal sitting-room. It does not seem very clear how the corridor is lighted. On the north side of it are the bathrooms and closets, and again we notice that the latter are not cut off by ventilating passages. The upper floors are similar to the ground floor.

Over one part of the out-patients' department are the male and the female accident wards, the former containing four beds, the latter two. The cross-ventilation of the male ward can hardly be first rate, and the female ward has no cross-ventilation at all except what it may get from a bay window and a probable fanlight over the door.

Over the lecture hall and museum are the operating theatre and the anæsthetic room. The analysis room, photographic room, and dark room find their places over the matron's rooms. The officers' bedrooms are placed on the first floor of the administrative block.

One of the basement floors contains dining-rooms for the staff, and there is a room for Swedish gymnastics, while even Turkish, Russian, and electric baths have not been forgotten. These things show how well abreast, or rather how far ahead, the hospital is of the most of its fellows.

The whole building is wonderfully compact, and the available land has been most carefully utilised. The total number of beds is 220, and, as already stated, the cost was £120,000.

The architects were Messrs. Pennington, of London, and Mr. C. W. Harvey, of Liverpool. Messrs. Thornton and Sons were the contractors.

THE DAVID LEWIS NORTHERN HOSPITAL, LIVERPOOL.

FIRST FLOOR PLAN.

## Figures and Tables

**Figure f1:**
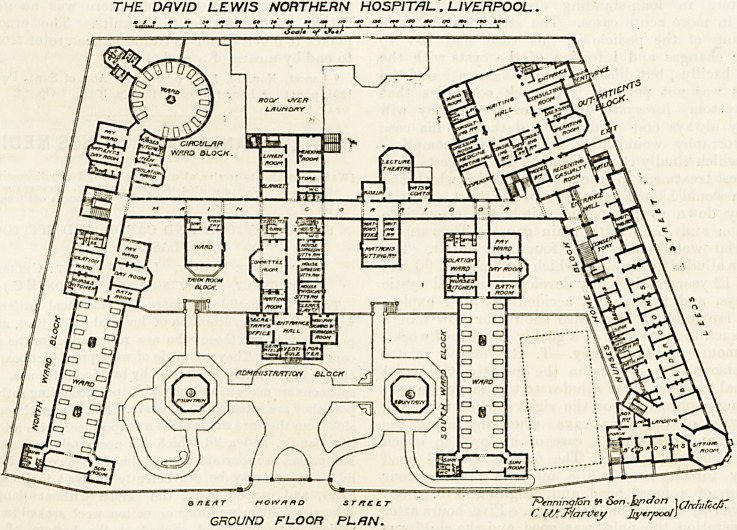


**Figure f2:**